# Evolution in the management of thyroid surgery over a period of 15 years in a Belgian center

**DOI:** 10.1186/s12893-024-02471-7

**Published:** 2024-06-15

**Authors:** Clotilde Saïe, Victor Marchand, Jules Zhang-Yin, Vincent Ers, Etienne Mauel

**Affiliations:** 1 Department of Endocrinology, Clinique du Sud Luxembourg, Arlon, Belgium; 2 Department of Surgery, Clinique du Sud Luxembourg, Arlon, Belgium; 3 Department of Nuclear Medicine, Clinique du Sud Luxembourg, Arlon, Belgium; 4 Vivalia Arlon, avenue des déportés 137, Arlon, 6700 Belgium

**Keywords:** Thyroid cancer, Thyroid surgery, Fine-needle aspiration, Thyroid weight, Thyroid recommendation

## Abstract

**Background:**

Guidelines for thyroid surgery have evolved to reflect advances in medical knowledge and decrease the overdiagnosis of low-risk thyroid cancer. Our goal was to analyze the change made in operative thyroid management and the impact on thyroid cancer diagnosis.

**Background:**

Guidelines for thyroid surgery have evolved to reflect advances in medical knowledge and decrease overdiagnosis of low risk thyroid cancer. Our goal was to study the evolution, over a long period, of pre- and postoperative management and the influence on histological cancer diagnosis and, more particularly, microcancer.

**Methods:**

In this retrospective cohort study, we included 891 consecutive patients who underwent thyroid surgery between 2007 and 2020.

**Results:**

Respectively 305, 290 and 266 patients underwent surgery over the 3 periods of 2007–2010, 2011–2015 and 2016–2020. In all three periods, women represented approximately 70% of the population, and the mean age was 54 years old (range: 67). Most surgeries (90%) involved total thyroidectomies. Over the study period, the proportion of preoperative fine needle aspiration (FNA) increased from 13 to 55%, *p* < 0,01. Cancer was found in a total of 116 patients: 35 (11%) patients between 2007 and 2010, 50 (17%) between 2011 and 2015 and 32 (12%) between 2016 and 2020 (*p* = 0.08). For all 3 periods, papillary thyroid cancer (PTC) was the most prevalent, at approximately 80%. The proportion of thyroid cancer > T1a increased significantly from 37% (2011–2015 period) to 81% (2016–2020 period), *p* = 0.001. Patients treated with radioiodine remained relatively stable (approximately 60%) but were more frequently treated with a low dose of radioiodine (*p* < 0.01) and recombinant human TSH (*p* < 0.01). Operative thyroid weight decreased over time in all but the low-risk T1a PTC cases.

**Conclusions:**

Over a period of 15 years and according to the evolution of recommendations, the care of patients who underwent thyroid surgery changed with the increased use of preoperative FNA. This change came with a decrease in low-risk T1a PTC.

## Background

An increase in the incidence of thyroid cancer has been reported worldwide [1–4]. Several studies have demonstrated a link between the rising incidence of thyroid cancer and the increasing use of medical imaging [5, 6]. This increase in incidence was mainly due to the rise in the number of papillary thyroid cancer (PTC) cases but was not associated with an increase in mortality [1, 2]. Indeed, low-risk PTC (T1a PTC) in particular is known to have a negligible impact on survival.

In Belgium, a previous study demonstrated geographical thyroid cancer incidence variation between the 2 major regions: Flanders and Wallonia [7]. This variation was mainly due to low-risk PTC and was paralleled by differences in clinical practice. For example, in Wallonia, a high incidence region (HIR), less thyroid surgery was preceded by fine needle aspiration (FNA) [8]. One study also showed a negative correlation between thyroid specimen weight and T1a PTC incidence, with possible overdiagnosis [9].

European and American recommendations for thyroid surgery have evolved over the years, as new research and clinical experience have provided a better understanding of thyroid cancer and its management [10, 11]. Guidelines have shifted toward more conservative approaches and emphasize the importance of risk stratification to avoid unnecessary surgery and decrease overdiagnosis of low-risk PTC. They underline the importance of preoperative FNA in the evaluation of thyroid nodules. Indeed, the use of FNA before surgery allows better patient selection for surgery. However, information about local adherence to international guidelines and evolution over time has been lacking.

The postoperative management of thyroid cancer has also changed with a more restrictive postoperative use of radioactive iodine (RAI) [10, 11]. The recommended dose has decreased, and today, preparation with recombinant human thyroid-stimulating hormone (rhTSH) is more frequently used [11].

Our goal was to study the evolution, over a long period, of pre- and postoperative management and the influence on histological cancer diagnosis and, more particularly, microcancer.

## Method

A retrospective observational cohort study was conducted. All consecutive patients who underwent thyroid surgery (total thyroidectomies or lobectomies) in our hospital (Clinique du Sud Luxembourg, Vivalia, Arlon, Belgium) between January 1st, 2007, and December 31st, 2020, were included. Patients were identified via surgery records. The medical files of the selected patients were examined in detail. The standard patient characteristics (age, sex) and imaging exams (US, thyroid scintigraphy with iodine^123^ or technectium^99^, fine needle aspiration (FNA)) performed before surgery were collected. The main indication for surgery were based on medical records and divided into suspicious cytology (preoperative FNA with Bethesda from 3 to 6), thyrotoxicosis (TSH under laboratory range), nontoxic nodular thyroid disease (nodes > 3 cm or symptomatic), symptomatic goiter (goiter associated with dysphagia, dyspnea or cervical discomfort), multinodular goiter (asymptomatic goiter with at least one node between 1 and 3 cm) or other (none of the above indications). In the case of multiple indications, the first indication named on this list was retained. The pathology reports were reviewed to extract information on thyroid weight specimens, histology results, exact tumor size and TNM stage. FNA was categorized according to the Bethesda system [12]. Postoperative data regarding radioiodine treatment were also obtained. Radioiodine treatments were performed at St Elisabeth Hospital (CHU, UCL, Namur, Belgium) and in our hospital. Patients were prepared with 4 weeks of thyroxine withdrawal or recombinant human thyroid-stimulating hormone (rhTSH), Thyrogen®. For follow-up, first cervical US results and the measurement of thyroglobulin performed between 6 and 12 months after surgery were recorded.

Patients were categorized according to the date of surgery. In the case of 2 surgeries (lobectomy and total thyroidectomy), the date of the first surgery was used. Group years were determined according to the publication of international guidelines in 2009 and 2015 [10, 11]. The implementation of guideline changes was discussed in departmental meetings and during. provincial endocrinologist conferences, with each practitioner also engaging in continuous education through attending various professional congresses.

The 3 groups were:

- Earliest period (EP): 2007–2010: between January 1st, 2007 and december 31st, 2010.
- Middle period (MP): 2011–2015: between January 1st, 2011 and december 31st, 2015.
- Latest period (LP) 2015–2020: between January 1st, 2016 and december 31st, 2020.

### Statistical analysis

JMP Pro 16.0.0 software was used for all the analyses. Continuous variables are expressed as medians and ranges, and categorical variables are expressed as counts and percentages. Categorical variables were analyzed using the chi-squared test or Fisher’s exact test. Continuous variables were analyzed using the Wilcoxon rank sum test. The Pearson correlation coefficient was used to determine links between variables. *P* values < 0.05 were considered significant.

This study was ethically approved by the ethical committee of Clinique du Luxembourg (264 OM, 149), Vivalia, Arlon, Belgium. Given the retrospective nature of this study, this ethical committee granted an exemption from requiring informed consent. The datasets used and analyzed during the current study are available from the corresponding author upon reasonable request.

## Results

### All patients

Our cohort included 891 patients, of whom 305 underwent surgery between 2007 and 2010, 290 between 2011 and 2015, and 266 between 2016 and 2020 (Table 1). For all periods, approximately two-thirds of the patients were female (*p* = 0.24). The median age was 53 years old (range: 75). Most of the surgeries performed were total thyroidectomies (90%).

**Table 1:** Characteristics, preoperative investigations and histological results in all cohort

	All patients				Thyroidectomies				Lobectomies			
	2007–2010	2011–2015	2016–2020	*p*	2007–2010	2011–2015	2016–2020	*p*	2007–2010	2011–2015	2016–2020	*p*
**n =**	305	290	266		277	263	239		28	27	27	
**Women. n (%)**	222 (73%)	204 (70%)	204 (77%)	0.2366	203 (73%)	187 (71%)	188 (79%)	0.1406	19 (68%)	17 (63%)	16 (60%)	0.8019
**Median age; range**	54; 67	53; 75	53; 64	0.8535	55; 65	54; 69	54; 64	0.8559	43.5; 61	45; 55	50; 44	0.6446
**Main indication for surgery**												
Thyrotoxicosis	46 (15.08%)	50 (17.24%)	58 (21.8%)	0.1003	43 (15.52%)	48 (18.25%)	55 (23.11%)	0.0862	3 (10.71%)	2 (7.4%)	3 (11.11%)	0.8804
Suspicious Cytology	19 (6.23%)	35 (12.07%)	46 (17.36%)	**0.0002**	19 (6.86%)	33 (12.55%)	44 (18.07%)	**0.0005**	0	2 (7.4%)	3 (11.11%)	0.2139
Symptomatic Goiter	41 (13.44%)	45 (15.52%)	24 (9.06%)	0.0685	41 (14.80%)	44 (16.73%)	22 (9.66%)	0.0632	0	1 (3.7%)	1 (3.70%)	NA
NT nodular disease	118 (38.69%)	122 (42.07%)	97 (36.60%)	0.4097	96 (34.66%)	101 (38.40%)	78 (32.35%)	0.3563	22 (78.57%)	21 (77.78%)	20 (74.07%)	0.9155
Mutlinodular goiter	66 (21.64%)	22 (7.59%)	31 (11.70%)	**< 0.0001**	66 (23.83%)	22 (8.37%)	31 (13.03%)	**< 0.0001**	0	0	0	
Other	15 (4.92%)	16 (5.52%)	9 (3.40%)	0.477	12 (4.33%)	15 (5.70%)	9 (3.78%)	0.5682	3 (10.71%)	1 (3.7%)	0	0.172
**Preoperative investigations**												
Ultrasound	269 (88.2%)	274 (94.8%)	254 (95.5%)	**0.0009**	244 (88.1%)	247 (94.3%)	227 (95%)	**0.0046**	25 (89.3%)	27 (100.00%)	27 (100.00%)	**0.0497**
Scintigraphy	222 (73%)	187 (64.7%)	198 (74.4%)	**0.0224**	199 (72.1%)	171 (65.3%)	180 (75.3%)	**0.0396**	23 (82.1%)	16 (59.3%)	18 (66.7%)	0.1695
Cytology	41 (13.5%)	88 (30.3%)	146 (55.1%)	**< 0.0001**	35 (12.7%)	75 (28.5%)	123 (51.7%)	**< 0.0001**	6 (21.4%)	13 (48.1%)	23 (85.2%)	**< 0.0001**
Bethesda (3–4)	13 (4.3%)	26 (9%)	42 (15.8%)	**< 0.0001**	13 (4.7%)	24 (9.1%)	37 (15.5%)	**0.0002**	0	2 (7.4%)	5 (18.5%)	**0.0473**
Bethesda (5–6)	5 (1.6%)	10 (3.45%)	16 (6%)	**0.019**	5 (1.8%)	10 (3.8%)	16 (6.7%)	**0.0172**	0	0	0	NA
Bethesda [3–6]	18 (5.9%)	36 (12.4%)	58 (21.9%)	**< 0.0001**	18 (6.5%)	34 (13%)	53 (22.3%)	**< 0.0001**	0	2 (7.4%)	5 (18.5%)	**0.0473**
**Histological results**												
Median thyroid Size ; range	46; 472	37; 300	33; 273	**0.0006**	49; 271	39; 300	34.5; 273	**0.0001**	21; 38	22; 48	20.5; 98	0.7794
Histological cancer n (%)	35 (11.5%)	50 (17.2%)	32 (12%)	**0.0839**	35 (12.7%)	49 (18.6%)	32 (13.4%)	0.1115	0	1 (3.7%)	0 (0%)	NA
Histological cancer > T1a	13 (37.1%)	23 (46%)	25 (80.6%)	**0.0009**	13 (37.1%)	22 (45%)	25 (80.6%)	**0.0008**	0	1 (100%)	0 (0%)	NA

The use of preoperative ultrasound was widespread and increased to up to 95% in the last period (*p* = 0.009). Over the 3 time periods, the use of FNA also increased significantly from 13.5% in the EP to 55% in the LP (*p* < 0.01). Likewise, there was an increase in the proportion of suspicious cytology (Bethesda 3 to 6), which also increased from 6 to 22% (*p* < 0.01).

Over the study period, the proportion of patients operated on for a multinodular goiter significantly decreased (from 21 to 11%, *p* < 0.01). On the other hand, the indication for surgery for suspicious nodules increased from 6 to 17% (*p* < 0.01).

Thyroid cancer was found in 11%, 17% and 12% of patients, respectively.

### Patients with thyroid cancer

A total of 116 cancers were histologically described between 2007 and 2020 (Table 2). Almost all patients underwent total thyroidectomies. The proportion of preoperative FNA increased from 26 to 71% (*p* = 0,005). The proportion of suspicious cytology (Bethesda 5 and 6) also increased significantly (*p* = 0,02). Regarding histology, one was a lymphoma, and 4 were anaplastic cancers. PTC was the most prevalent throughout the periods (Table 3). The median size of the DTC was 12 mm (range: 74.5). The median size increased to up to 17 mm (range: 42.5) at the LP (*p* = 0.0079). The proportion of T1 was 60% at the EP, 54% at the MP and 19.35% at the LP (*p* = 0.0016). Regarding thyroid cancer > T1a only, the proportion increased over the study period from 37 to 80% (*p* = 0.001). Radioiodine was administered to a total of 63 patients. Notably, the median dose decreased at the LP to 30 mGy (*p* < 0.001). The proportion of preparation with rhTSH increased over the periods. For pT1a patients, the decrease in RAI was marked. Regarding follow-up, the monitoring of thyroglobulin was prevalent (83% at the LP), and US was performed in approximately one-third of patients over the 3 periods (*p* = 0.94).

**Table 2:** Preoperative phase in thyroid cancer patients

	2007–2010	2011–2015	2016–2020	*p*
**n =**	35	50	31	
**women. n (%)**	22 (62.86)	31 (62)	25 (80.65)	0.1775
**Age median. range**	53. 59	52. 69	54. 50	0.9715
**Preoperative investigations**				
Ultrasound	33 (94.29%)	48 (97.96%)	31 (100%)	0.3294
Scintigraphy	24 (68.57%)	29 (59.18%)	23 (74.19%)	0.3593
Cytology	9 (25.71%)	18 (36%)	22 (70.97%)	**0.0005**
Bethesda (1)	1 (2.86%)	2 (4%)	2 (6.45%)	0.7651
Bethesda (2)	1 (2.86%)	3 (6%)	1 (3.22%)	0.7358
Bethesda (3–4)	3 (8.57%)	6 (12%)	8 (25.81%)	0.1108
Bethesda (5–6)	4 (11.43%)	7 (14%)	11 (35.48%)	**0.0224**
**Type of surgery**				
Thyroidectomy	35 (100%)	49 (98%)	31 (100%)	0.5139
Lobectomy	0 (0%)	1 (2.63%)	0 (0%)	NA

**Table 3:** Postoperative phase in thyroid cancer patients

	2007–2010	2011–2015	2016–2020	*p*
**n =**	35	50	31	
**Thyroid volume median; range**	44; 138	34; 188	30; 263	0.0735
**Histology**				
Papillary classical variant	13 (37.14%)	17 (34%)	15 (49.39%)	0.422
Papillary follicular variant	17 (48.57%)	24 (48%)	11 (35.48%)	0.4733
Follicular	0 (0%)	4 (8%)	2 (6.45%)	0.2432
Anaplastic	1 (2.86%)	2 (4%)	1 (3.23%)	0.9574
Medullary	4 (11.43%)	1 (2%)	0 (0%)	**0.0419**
Others	0 (0%)	2 (4%)	3 (6.45%)	0.3438
**Cancer size. median; range**	10; 69.5	7; 74.5	17; 42.5	**0.0079**
**TNM**				
T1a	21 (60%)	27 (54%)	6 (19.35%)	**0.0016**
T1b	4 (11.43%)	9 (18%)	10 (32.26%)	0.0967
T2	8 (22.86%)	6 (12%)	12 (38.71%)	**0.0197**
T3	1 (2.86%)	6 (12%)	3 (6.68%)	0.3255
T4	0 (0%)	1 (2%)	0 (0%)	0.5139
N1	5 (14.29%)	5 (10%)	2 (6.45%)	0.5772
**RAI**				
Patients treated by RAI n (% of DTC patients)	17 (57%)	22 (49%)	21 (70%)	0.3686
Median dose; range	100; 50	100; 120	30; 85	**< 0.0001**
Thyroxin withdrawal	15 (88%)	9 (40%)	3 (14%)	**0.0242**
rhTSH	2 (12%)	13 (60%)	18 (86%)	**< 0.0001**
**Radioiodine in pT1a**				
All pT1a patients ; n	21	27	6	
T1a patients treated by RAI n(%)	6 (28%)	4 (14%)	0	
Median dose (mCi); range	108 ; 50	82.5 ; 70	-	
Thyroxin withdrawal	5	1	-	
rhTSH	1	3	-	
**Follow-up**				
Post operative scintigraphy	19 (63.33%)	34 (73.91%)	28 (93.33%)	**0.0205**
Neck US	11 (36.67%)	15 (34.09%)	11 (37.93%)	0.9407
Thyroglobulin	21 (70%)	32 (69.57%)	25 (83.33%)	0.3563

RAI: radioiodine

rhTSH: recombinant human thyroid-stimulating hormone

### Size of thyroid specimen (table 4)

**Table 4:** Evaluation of thyroid weight

	All patients (thyroidectomies)					Excluded thyrotoxicosis				
	2007–2010	2011–2015	2016–2020		2007–2010		2011–2015		2016–2020	
**n =**	255	255	235		213		208		181	
**Thyroid weight known n=**	277	262	240		234		214		184	
**Median Thyroid weight**	49	39	34		45		37		33	
**Range**	471	300	273		471		300		176	
	**DTC patients**			**PTC pT1a patients**						
	**2007–2010**	**2011–2015**	**2016–2020**		**2007–2010**		**2011–2015**	**2016–2020**		
**n =**	28	47	30		16		27	6		
**Thyroid weight known n=**	36	48	30		21		27	6		
**Median Thyroid weight**	44,5	33	30		53,5		33	51		
**Range**	138	188	263		118		130	51		

In all cohorts, the median thyroid weight in patients who underwent total thyroidectomy was 39 g (range: 476) and decreased from 49 g (range: 471) at the EP to 34 g (range: 273) at the LP (*p* = 0,01). This decrease persisted after excluding those patients who had surgery for thyrotoxicosis (45 g (range: 471) at EP to 33 g (range: 176) for LP). Regarding the patients with cancer, thyroid volume decreased from 44.5 g (range: 138) to 30 g (range: 263), but the difference was not significant (*p* = 0,07). This decrease was not found in the patients with PT1a PTC: the thyroid weight remained stable (53 g at the EP and 51 g at the LP).

## Discussion

The objective of our study was to analyze the surgical cohort over 3 time periods after the publication of international guidelines, specifically the American Thyroid Association Guidelines of 2009 and 2015 [10, 11]. Over the years of our study, with modifications in practice, such as an increased use of FNA, the proportion of patients with low-risk cancer decreased.

Worldwide, an increasing incidence of thyroid cancer has been observed, mainly due to low-risk papillary thyroid cancer [2–5]. In Belgium, a retrospective population-based cohort study demonstrated regional variation between the North of the country (Flanders), with a low incidence, and the South part (Wallonia), with a higher incidence [7]. These differences were most marked for low-risk disease and were associated with different practices in thyroid imaging and surgery. The increase in low-risk cancer could be considered a consequence of overdiagnosis and possibly overtreatment.

To limit overdiagnosis, FNA has taken a major part. In 2009 guidelines [10], the use of FNA was proposed for all nodules > 1 cm. The 2015 guidelines [11] confirmed that FNA remains the gold standard technique in the evaluation of thyroid nodules. In our cohort, the use of FNA in cancer thyroid patients significantly increased from 25% (before publication of 2009 guidelines) to 71% after 2016, confirming the adoption of the new recommendation. In the study conducted by Decallone et al., the use of FNA differed from 35.3% in HIR to 70.2% in regions with lower incidence (LIR) [8]. This variation was also reported in France, with a variation between regions ranging from 11 to 53% [13]. There are probably several causes for the delay in the implementation of FNA: latency in the diffusion of recommendations and the modification of the standard of care, the availability of doctors able to perform this technique and cytopathology, a low reimbursement rate… In our study, as in the literature, the increase in FNA use was concomitant with the decrease in the incidence of micro cancer from 60 to 19%. Unfortunately, in some cases cytology cannot accurately discriminate malignant from benign nodules, with a malignancy risk between 5 and 35% (Bethesda classification III and IV). In this case, molecular testing to evaluate FNA samples has been proposed since 2009 and emphasized in 2015. This technique is not available in our center and has not been studied.

Another advancement during the study period concerns the evolution of diagnostic imaging techniques, such as improved ultrasound resolution, elastography, and molecular imaging. However, our study could not address this due to the variability of radiologists and machines used over the years, making it impossible to study this criterion, particularly since elastography was not universally performed.

Thyroid surgery represents the first line therapeutic approach for thyroid cancer [14]. Total thyroidectomy was first widely used. In 2009, lobectomy was proposed for suspicious nodules < 1 cm. In 2015, lobectomy was also proposed for suspicious nodules from 1 to 4 cm without extrathyroidal extension and without clinical evidence of any lymph node metastases, as thyroid lobectomy alone may be sufficient initial treatment for low-risk papillary and follicular carcinomas [11, 15]. In our center, this trend was not retrieved. Outside of central habits, it is possible that in our iodine-deficient region (Ardennes massif), contralateral nodules have often been found. How to improve the large-scale diffusion of guidelines remains a significant issue [16]. Even more with new perspectives, such as the development of active surveillance for small PTC or minimally invasive treatment, which will probably once again change recommendations for thyroid cancer [17, 18].

Recent guidelines also focused on postoperative management with a de-escalation of treatment and, more particularly, a decrease in the use of RAI [11, 19]. Accordingly, we found a decrease in the doses of RAI, as in other studies. In a review in the USA [20], after 2015, a decrease in RAI was particularly marked for PTC < 4 cm. Several studies have shown significant variation in RAI use, reflecting a difference in adherence to newly published guidelines. Various factors have been described, such as physician specialty, patient preference, type of hospital, and concern for cancer progression [21–23]. Regarding follow-up, the use of thyroglobulin has long been established. However, there was notably insufficient use of cervical US.

In the study conducted by Decallone et al., thyroid weight was proposed as a marker for the thyroid surgical threshold [8]. This study reported a 30% higher thyroid weight in the LIR group than in the HIR group (from 34.5 g to 27.3 g). In all patients and in DTC patients, thyroid weight tended to decrease over time. For the last period, in DTC patients, thyroid weight averaged 30 g, between the HIR (27.5 g) and the LIR (36 g), previously described. Notably, this decrease was not found for patients with PT1a PTC (53 g at the EP and 51 g at the LP). This could be due to a difference in iodine intake in our region near the Ardennes mountain range, where there is a higher rate of goiter and occult microcancer [24, 25]. Literature data regarding surgical thyroid specimen weight are sparse, and more studies are necessary to implement this threshold for thyroid surgery.

Our study has several limitations. One notable limitation of our study is its retrospective nature, which inherently restricts our ability to establish a causal relationship between the guideline changes and the observed outcomes. Furthermore, the evaluation of indications or symptoms could present a bias. Another limitation of our study is its single-center design, which may restrict the generalizability of our findings. Addressing these limitations would require prospective studies with broader geographic representation. Time periods were selected according to the publication of international guidelines in 2009 [10] and 2015 [11] and were not completely homogeneous (4 years for the EP and 5 years for other periods). Our study specifically focused on initial management and histopathological outcomes, and did not include long-term patient outcomes such as recurrence rates and survival. Future studies should address these aspects to provide a more comprehensive evaluation of the impact of management changes. This observational study provides an overview of all thyroid surgeries and not exclusively of thyroid cancer. It also raises interest regarding the diffusion of recommendations in real life.

## Conclusion

In conclusion, over time and according to the evolution of recommendations, the care of patients who underwent thyroid surgery changed, particularly with the increased use of preoperative FNA. This change came with a decrease in the proportion of low-risk T1a PTC.

## Data Availability

The datasets used and analyzed during the current study are available from the corresponding author upon reasonable request.
